# Dynamic heterogeneity and non-Gaussian statistics for acetylcholine receptors on live cell membrane

**DOI:** 10.1038/ncomms11701

**Published:** 2016-05-26

**Authors:** W. He, H. Song, Y. Su, L. Geng, B. J. Ackerson, H. B. Peng, P. Tong

**Affiliations:** 1 Nano Science and Technology Program, Hong Kong University of Science and Technology, Clear Water Bay, Kowloon, Hong Kong; 2 Department of Physics, Hong Kong University of Science and Technology, Clear Water Bay, Kowloon, Hong Kong; 3 Division of Life Science, Hong Kong University of Science and Technology, Clear Water Bay, Kowloon, Hong Kong; 4 Department of Physics, Oklahoma State University, Stillwater, Oklahoma 74078, USA

## Abstract

The Brownian motion of molecules at thermal equilibrium usually has a finite correlation time and will eventually be randomized after a long delay time, so that their displacement follows the Gaussian statistics. This is true even when the molecules have experienced a complex environment with a finite correlation time. Here, we report that the lateral motion of the acetylcholine receptors on live muscle cell membranes does not follow the Gaussian statistics for normal Brownian diffusion. From a careful analysis of a large volume of the protein trajectories obtained over a wide range of sampling rates and long durations, we find that the normalized histogram of the protein displacements shows an exponential tail, which is robust and universal for cells under different conditions. The experiment indicates that the observed non-Gaussian statistics and dynamic heterogeneity are inherently linked to the slow-active remodelling of the underlying cortical actin network.

## Abstract (web summary)

Molecular motion in living cells is known to be more complicated than those determined solely by thermal equilibrium, but a quantitative analysis is still missing. Here, He *et al*. quantify the lateral motion of proteins on live muscle cell membranes, which doesn't follow the normal Brownian diffusion.

## Graphical abstract

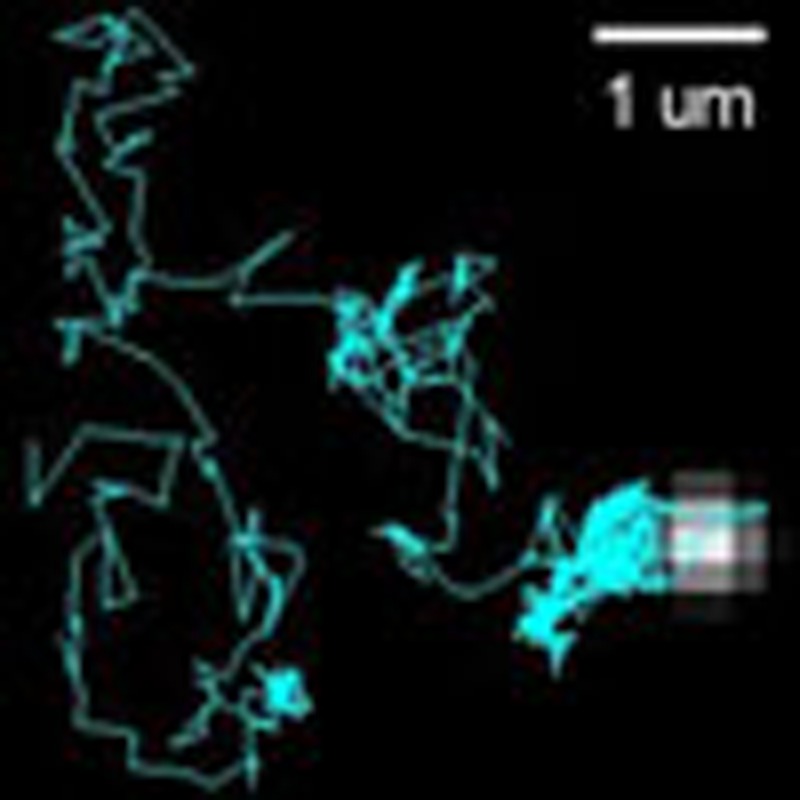

Cell membranes, which define cell boundaries and maintain communication with the outside world, display an intriguing array of structural complexes of lipids/cholesterols and various proteins essential to the existence and functioning of the cell. In the original fluid mosaic model^1^, the cell membrane was thought of as a quasi-two-dimensional fluid layer in which proteins are dispersed randomly at a low concentration and can float unencumbered. From the wealth of new data obtained in recent years, our general view of membrane architecture has evolved into a new paradigm in which the membrane has variable patchiness and thickness and a higher protein occupancy than previously thought^2^. The lipids and proteins on the membrane are not ideally mixed, and form molecular complexes ranging from nano-scale ‘lipid rafts'^3^,^4^ and protein clusters to micron-sized stable domains such as caveolae, microvilli and focal adhesions.

Moving in a structured membrane, the proteins do not enjoy continuous and unrestricted lateral diffusion as was originally envisioned^5^. Instead, proteins diffuse in a very complex landscape with considerable lateral heterogeneity in the membrane^6^,^7^. Transmembrane proteins also interact strongly with the underlying cytoskeletal cortex^4^. Using single-particle tracking (SPT) techniques^7^,^8^, one can directly observe and follow the motion of individual proteins. The measured protein trajectories have been found to be quite heterogeneous^6^,^7^,^9^, with some moving fast and appearing to diffuse freely while others are transiently confined to small membrane domains. A main issue in the continuing discussion is whether the dynamic heterogeneity of the transmembrane proteins is caused by the effect of clustering imposed by membrane clusters^3^,^10^, such as lipid rafts, or by membrane partitions generated by interactions with the underlying cortical actin network^4^, such as ‘membrane-skeleton fences'^7^,^11^,^12^. Most of the theoretical discussions assumed that membrane organization is governed by equilibrium processes, such as critical thermal fluctuations and ligand-binding equilibrium.

The available SPT data are not conclusive because the protein trajectories were sampled over a relatively short time (due to the finite lifetime of the florescent probes used), and thus heavily influenced by the surrounding molecules without revealing their long-time behaviour and their interactions with distant molecules on the membrane^13^. In addition, the current analysis of protein motion often focuses on identifying only a few targeted single molecular events, while ignoring other molecular events of possibly equal importance owing to the lack of systematic statistical analysis. Such analysis is extremely important, because stochastic fluctuations at the single molecular level are significant^14^. The lack of a systematic analysis of the protein motion is partially due to the fact that direct measurement of the statistical properties, such as the probability density function (normalized histogram or PDF) *P*(Δ*x*) of the protein displacement Δ*x*, often requires a large volume of individual protein trajectories, which are difficult to obtain from living cells. As a result, most previous studies in this area only measured the mean-squared displacement (the lowest moment of *P*(Δ*x*)), which requires less statistics but is not adequate to describe the complex motion of proteins in a living cell^15^.

In this paper, we report a systematic study of the lateral motion of a transmembrane protein on live muscle cell membranes cultured from *Xenopus* embryos. The protein chosen for the study is acetylcholine receptor (AChR), which is a well characterized neurotransmitter receptor for the study of neuromuscular junctions^16^,^17^. The lateral mobility of AChRs plays an essential role in determining the response of the postsynaptic membrane to neurotransmitter stimuli. The individual AChRs are labelled by bright and photostable fluorescent quantum dots (QDs). With the help of an advanced single-molecule tracking algorithm, we are able to obtain a significantly large volume of individual AChR trajectories from more than 360 live cells over a wide range of sampling rates (up to 80 Hz) and long durations (up to 200 s). A central finding of this investigation is that the moving trajectories of the individual AChRs do not follow the Gaussian statistics for normal Brownian diffusion. Instead, we show for the first time that the measured PDF *P*(Δ*x*) has an exponential tail, which is robust and universal for cells under different conditions. A theoretical model is developed to explain why the structurally identical AChRs have very different dynamic behaviours with an exponential-like distribution in their diffusion coefficient.

## Results

### Characterization of the AChR trajectories

In the experiment, we obtain the AChR trajectories from consecutive images of the QDs, and find their position **r**(*t*) (and hence the position of AChRs) at time *t* using a homemade SPT program with a spatial resolution of ∼20 nm. Because the viscosity of the plasma membrane is ∼1,000 times higher than that of the extracellular medium, the motion of the QD-labelled AChRs is determined primarily by their transmembrane domains^18^. From the AChR trajectories, we compute the statistics of the two-dimensional displacement vector, Δ**r**(*τ*)=**r**(*t*+*τ*)−**r**(*t*), over delay time *τ*, such as the mean-squared displacement (MSD) 〈Δ*r*^2^(*τ*)〉 and the PDF *P*(Δ*x*) of the *x*-component of Δ**r**. We also compute the radius of gyration *R*_g_ of the AChR trajectories *R*_g_^2^(*τ*)=(1/*N*)∑_*i*_^*N*^[(*x*_*i*_−〈*x*〉)^2^+(*y*_*i*_−〈*y*〉)^2^], where *N* is the total number of time steps in each trajectory, *x*_*i*_ and *y*_*i*_ are the projection of the position of each trajectory step on the *x*- and *y*-axis, respectively, and 〈*x*〉 and 〈*y*〉 are their mean values. Physically, *R*_g_ quantifies the size of an AChR trajectory generated during the time lapse *τ*.

**Figure 1:**
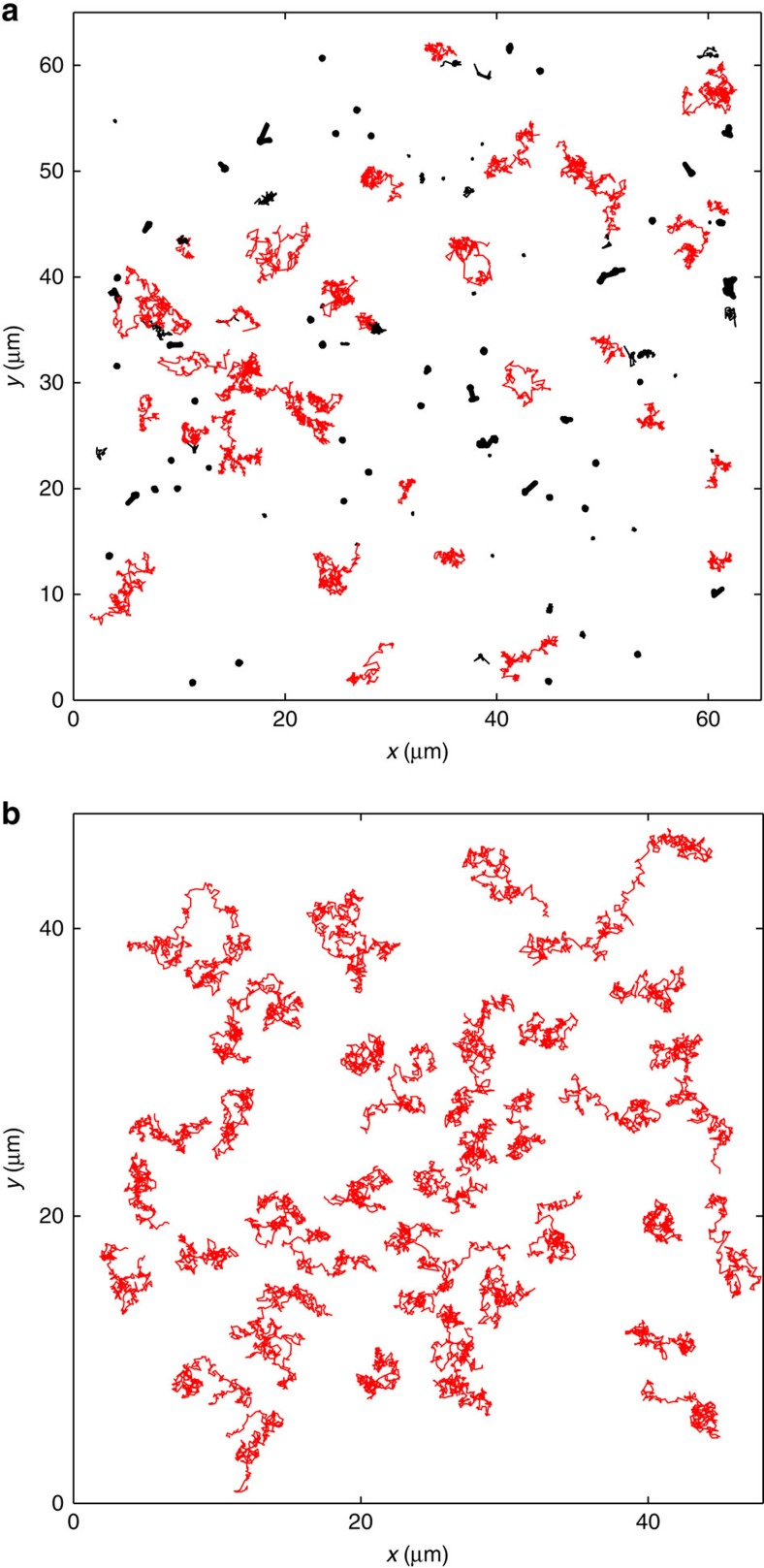
Observed dynamic heterogeneity in the AChR trajectories. (**a**) Overall 130 representative AChR trajectories with 300 time steps (60 s). These trajectories are taken from the bottom membrane of a *Xenopus* muscle cell. Red trajectories indicate fast moving AChRs and black ones indicate ‘nearly immobile' AChRs. (**b**) A total of 52 representative trajectories of silica spheres 2.14 μm in diameter undergoing Brownian diffusion in water over a flat substrate with 1,000 time steps (47 s).

Figure 1a shows a representative collection of 130 AChR trajectories over a time interval of 60 s. These identical AChRs exhibit a huge amount of dynamic heterogeneity as evidenced by the large variation in trajectory sizes; some being mobile (red trajectories) and others nearly immobile (black trajectories). Among the mobile AChRs, some move fast (with a large trajectory size) and others slower (with a smaller trajectory size). The situation shown in Fig. 1a is in great contrast with the Brownian motion of colloidal particles in a simple fluid, as show in Fig. 1b. The distribution of the Brownian trajectories is much more uniform than that of the AChR trajectories.

**Figure 2:**
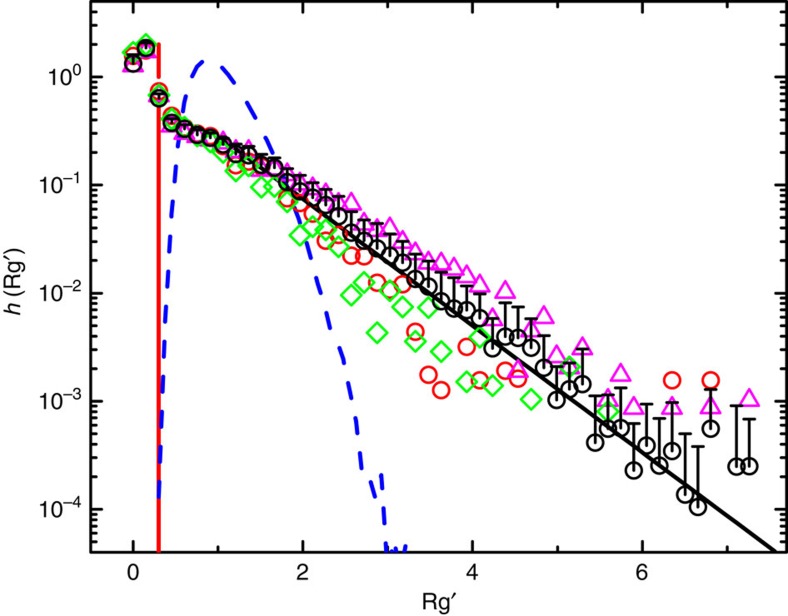
Normalized histogram of the radius of gyration of AChR trajectories. Measured PDF 
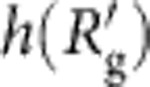
 of the normalized 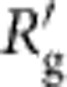 for the AChR trajectories taken under different sample conditions: cultured for 1 day after dissection (red circles), cultured for 4 days (magenta triangles), and cultured for 8 days (green diamonds). Each 
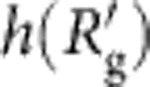
 is obtained by averaging the data from 10 cells cultured under the same condition. The black circles are obtained by averaging the data from 70 cells. Their statistics is considerably improved with small error bars indicating the standard deviations. The solid black line shows the exponential function, 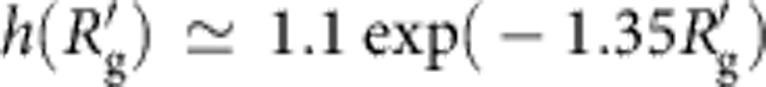. The dashed blue line shows the measured 
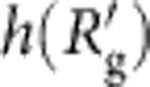
 for silica spheres undergoing Brownian diffusion. The vertical red line indicates the cutoff value 
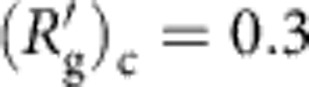
 used to define the immobile trajectories.

To have a quantitative description of the AChR trajectories, we calculate their normalized radius of gyration 
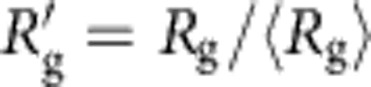
, where 〈*R*_g_〉 is the mean value of *R*_g_. For Brownian diffusion, one has *R*_g_(*τ*)=[(2/3)*D*_0_*τ*]^1/2^ with *D*_0_ being the diffusion coefficient (see Supplementary Note 1 for more details). For live cells, we define 〈*R*_g_〉=[(2/3)〈*D*_L_〉*τ*]^1/2^, where 〈*D*_L_〉 is the long-time diffusion coefficient averaged over 365 cells (see more discussions on Fig. 4 below). The use of the normalized 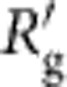 allows us to compare the AChR trajectories taken over different *τ* and/or under different sample conditions. Figure 2 shows the measured PDF (normalized histogram) 
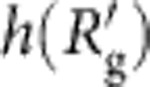
 of 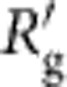 for the AChR trajectories taken under different sample conditions. All of the measured 
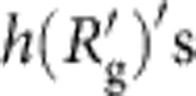
 collapse onto a single master curve, once the normalized 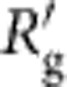 is used. The PDFs from different frogs and embryos and from cells cultured for different days and sampled at different *τ* exhibit a universal form (for clarity, some of the curves are not shown here). The measured 
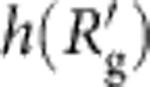
 has a peak at 
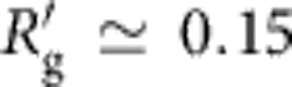
 followed by an exponential tail (black solid line). For silica spheres undergoing the Brownian motion, their 
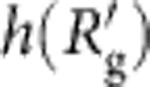
 has a narrow distribution peaked at 
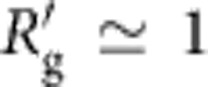
 (blue dashed line, see Supplementary Note 1 for more discussions). Figure 2 reveals that there are many fast moving AChRs, whose 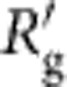 is larger than that for normal Brownian diffusion. The vertical red line indicates the cutoff value 
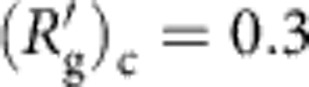
 used in the experiment, below which the AChR trajectories are treated as immobile ones (black trajectories in Fig. 1).

### Mean-squared displacement

**Figure 3:**
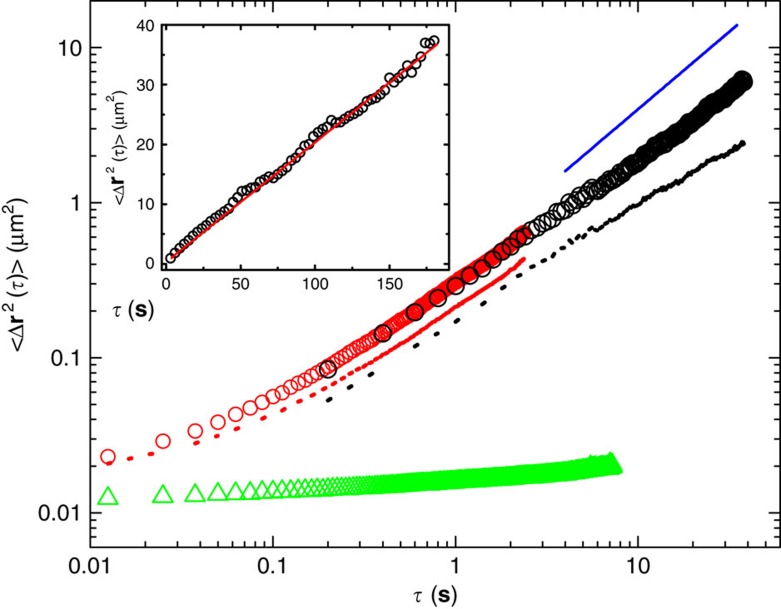
Crossover from sub-diffusion to normal diffusion observed from the MSD curve. Measured 〈Δ**r**^2^(*τ*)〉 as a function of delay time τ for the AChR trajectories taken at two sampling rates of 80 fps (red dashed line and circles) and 5 fps (black dashed line and circles). Data from a single cell are used in the ensemble average. The red and black dashed lines are obtained when both the mobile and immobile trajectories are included in the calculation. The red and black circles are obtained when only the mobile trajectories are included in the ensemble average. The green triangles are obtained when only the immobile trajectories are included in the ensemble average. The blue solid line indicates the relationship 〈Δ**r**^2^(*τ*)〉∼*τ* with a slope of unity in the log–log plot. Inset shows a linear plot of the measured 〈Δ**r**^2^(*τ*)〉 as a function of *τ* and the red solid line is a linear fit to the data points.

Figure 3 shows the measured MSD 〈Δ**r**^2^(*τ*)〉 as a function of *τ* for the AChR trajectories taken at two sampling rates of 80 and 5 frames per second (fps). The red and black dashed lines obtained at the two different sampling rates do not superimpose with each other in the common region of *τ* between 0.2 and 3 s. To achieve the higher sampling rate, the viewing area of the camera is cropped. Because of the spatial inhomogeneity of the immobile AChR distribution, we find the number ratio *γ* of the mobile AChR trajectories to the total number of trajectories obtained at the two sampling rates is different. Once the immobile trajectories are removed from the ensemble average, the measured 〈Δ**r**^2^(*τ*)〉 becomes reproducible and the two curves (red and black circles) superimpose well with each other. The final MSD curve (circles) in the log–log plot is not a linear function and goes as 〈Δ**r**^2^(*τ*)〉∼*τ*^*α*^ with 0.4<*α*<0.9 in the small-*τ* range 0.0125–1 s. Only at the long-time limit (*τ*>4 s), does the measured MSD become diffusive with *α*≃1 (blue solid line). In this case, 〈Δ**r**^2^(*τ*)〉≃4*D*_L_*τ*, where *D*_L_ is the long-time diffusion coefficient of the AChRs.

The long-time behaviour of 〈Δ**r**^2^(*τ*)〉 is best presented in the linear plot as shown in the inset of Fig. 3. Because of the high efficiency of our tracking algorithm (see Supplementary Methods for more details), we are able to obtain long-time trajectories of the AChRs with adequate statistics. About 1,160 samples are used to obtain 〈Δ**r**^2^(*τ*)〉 at the largest delay time *τ*≃160 s. The statistics for 〈Δ**r**^2^(*τ*)〉 at smaller values of *τ* are even better with the error bars smaller than the size of the symbols used in Fig. 3. In the wide range of *τ* between 4 and 160 s in which the AChRs have diffused more than 750 times of their own diameter, the measured 〈Δ**r**^2^(*τ*)〉 can be well described by a linear function of *τ* (red solid line). From the slope of the fitted solid line, we obtain *D*_L_=0.05 μm^2 ^s^−1^.

**Figure 4:**
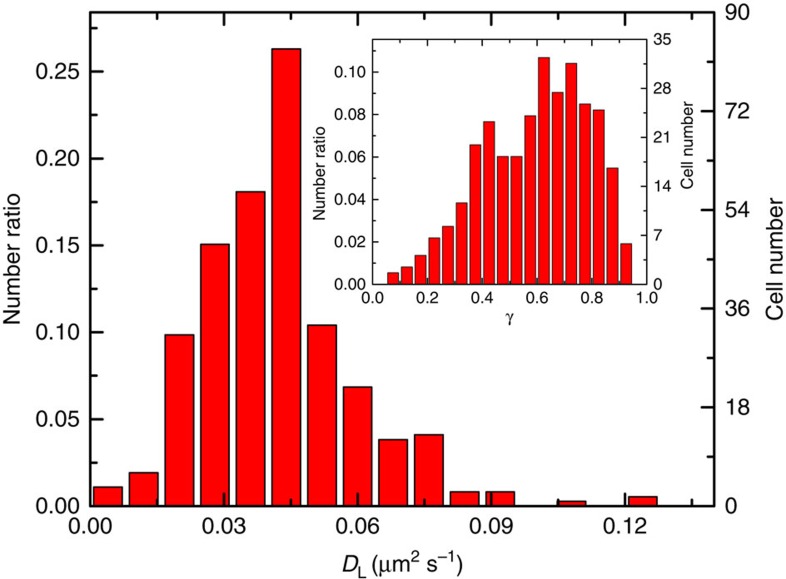
Cell-to-cell variations in the measured long-time diffusion coefficient *D*_L_ and mobile ratio γ of the AChRs. Distribution of the measured *D*_L_ of AChRs. Inset shows the distribution of the measured γ of the AChRs. The total number of cells used in the statistics is 365.

Figure 4 and its inset show, respectively, the final statistics of the measured *D*_L_ and the mobile ratio *γ* for the AChRs from different frogs and from cells cultured with different days. The value of *D*_L_ has a fairly narrow distribution with 〈*D*_L_〉=0.041±0.015 μm^2 ^s^−1^. The distribution of *γ* is broader with 〈*γ*〉=0.64±0.17. These mean values are obtained from 365 cells. The value of *γ* tends to be smaller for unhealthy cells and for cells cultured over a long period of time. We also examined the MSD curves obtained in different regions of the membrane in the same cell, both on the upper (away from the substrate) and lower (facing the substrate) portions of the membrane. The measured MSD in different regions remains approximately the same, suggesting that the AChRs on the same membrane have approximately the same value of *D*_L_, which can be used as a parameter to characterize the mobility of membrane proteins in living cells. Because the bottom portion of the membrane has a large planer view with more than 100 QDs in each frame, we used this imaging setup to collect more data for a better statistical analysis.

### Probability density function

**Figure 5:**
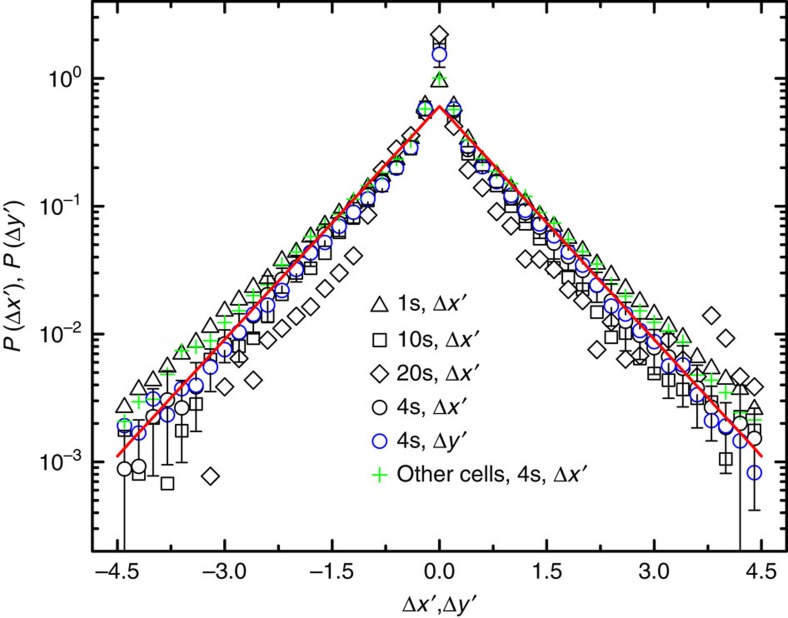
Non-Gaussian behaviour of the normalized histogram of AChR's displacements. Measured PDFs *P*(Δ*x*′) and *P*(Δ*y*′) of the normalized displacements Δ*x*′ and Δ*y*′ for the trajectories of mobile AChRs. Data are obtained from 10 cells under each sample condition: (i) Δ*x*′(*τ*) with *τ*=1 s (black triangles), 4 s (black circles), 10 s (black squares) and 20 s (black diamonds); (ii) Δ*y*′(*τ*) with *τ*=4 s (blue circles) and (iii) Δ*x*′(*τ*) with *τ*=4 s for a group of 10 cells from a different frog (green crosses). The red solid line is an exponential fit to the data points, *P*(Δ*x*′)≃*a* exp (−*β*|(Δ*x*′)|), with *a*=0.6 and *β*=1.4.

Another important quantity to characterize the motion of AChRs is the PDFs (normalized histograms), *P*(Δ*x*′) and *P*(Δ*y*′), of the *x*- and *y*- components of Δ**r**(*τ*) at a fixed value of τ. Here, Δ*x*′=Δ*x*/(2*D*_L_*τ*)^1/2^ and Δ*y*′=Δ*y*/(2*D*_L_*τ*)^1/2^ are the displacements normalized by the diffusion length (2*D*_L_*τ*)^1/2^. Figure 5 shows the measured *P*(Δ*x*′) and *P*(Δ*y*′) for mobile AChRs obtained under different sample conditions. Although the measured *D*_L_ varies considerably under different cell conditions, the PDFs collapse onto a single master curve, once the normalized Δ*x*′ (and Δ*y*′) is used. Except for a sharp peak near the origin, all of the PDFs have an exponential tail (red solid line). The error bars show the standard deviation of the black circles averaged over 10 cells. Because of the reduced number of data points, the diamonds have relatively larger experimental uncertainties. Figure 5 thus reveals that AChRs have a heavy-tailed distribution in their mobility, and this distribution is universal among the cells under different sample conditions. Similar *P*(Δ*x*′) (and *P*(Δ*y*′)) are also found for the AChRs on the upper portion of the membrane away from the substrate (see Supplementary Fig. 7 for more details). Evidently, the exponential PDF is a leptokurtic distribution, which has a higher peak and a heavier tail compared with the Gaussian PDF^19^.

### Immobile trajectories and statistical sampling conditions

Figure 3 reveals that the measured 〈Δ**r**^2^(*τ*)〉 under two different sampling conditions (red and black dashed lines) has different values in the common region of *τ*. We find that the immobile trajectories have a dominant role in determining the value of 〈Δ**r**^2^(*τ*)〉. The measured 〈Δ**r**^2^(*τ*)〉 for the immobile AChRs (green triangles) is about two orders of magnitude smaller than the value of 〈Δ**r**^2^(*τ*)〉 in the long-time regime (*τ*⪞4 s) for the mobile AChRs (black circles) and thus contributes many near-zero values to the ensemble average. At the higher sampling rate (80 fps), the viewing area of the camera is cropped and the number of immobile trajectories recorded in the movie becomes different from that obtain at the lower sampling rate (5 fps). This is caused by the spatial inhomogeneity of the immobile AChR distribution. As shown in Fig. 3, once the immobile trajectories are removed from the ensemble average, the measured 〈Δ**r**^2^(*τ*)〉 under two different sample conditions becomes identical in the common region of *τ* (red and black circles).

**Figure 6:**
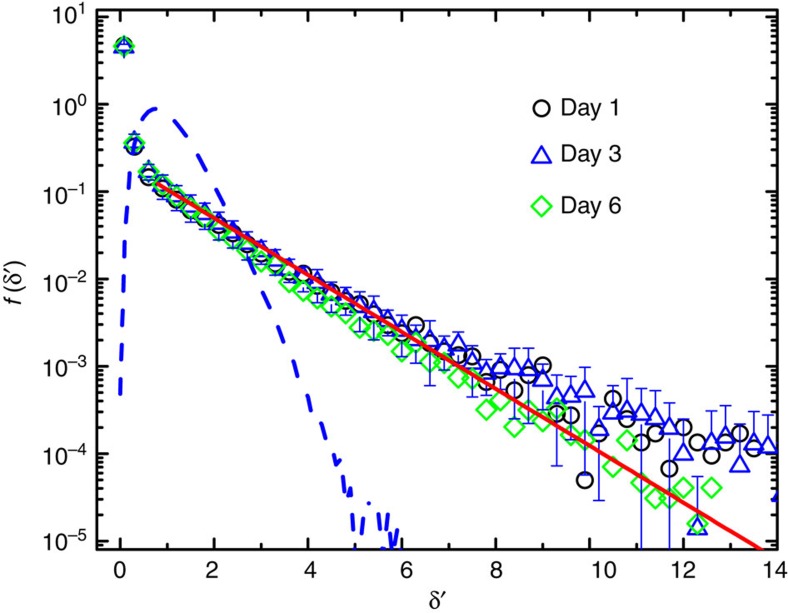
Normalized histogram of the ‘instantaneous' diffusion coefficient of mobile AChRs. Measured PDF *f*(*δ*′) of the normalized diffusion coefficient *δ*′=*δ*/*D*_L_ for the mobile AChR trajectories. Data are obtained from three groups of 10 cells each cultured for (i) 1 day (black circles), (ii) 3 days (blue triangles) and (ii) 6 days (green diamonds). The error bars show the standard deviation of the blue triangles averaged over 10 cells. The red solid line is an exponential fit to all data points, *f*(*δ*′)≃0.22 exp (−0.75*δ*′). The blue dashed line shows the measured *f*(*δ*′) for the silica spheres undergoing normal Brownian diffusion.

By carefully examining the AChR trajectories, we also find that even the mobile trajectories still have some immobile segments of varying lengths (durations). As shown in the Supplementary Movie, the AChRs often move for a while and are transiently confined to a small region on the membrane for a different amount of time (up to seconds) and then move again. The transient confinement of AChRs is also reflected in the measured 
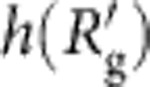
 in Fig. 2, *P*(Δ*x*′) in Fig. 5 and PDF *f*(*δ*) of the ‘instantaneous' diffusion coefficient *δ* in Fig. 6 below. For a fixed delay time *τ*, the immobile segments in a mobile trajectory tend to have smaller values of 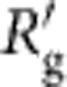, Δ*x*′, and *δ* and hence give rise to a peak in the corresponding histograms at small values of 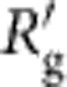, Δ*x*′ and *δ*. We believe that the transient confinement of AChRs is caused by the transient binding of the AChRs to the underlying cortical actin network. A similar effect was also observed for the Kv21 channel proteins in HEK 293 cells^20^.

The above observation of non-reproducible 〈Δ**r**^2^(*τ*)〉 resulting from different sampling of the immobile AChR trajectories may shed light on the problem of nonergodicity between the time- and space-averaged MSDs, which has been observed for a number of molecules in live cells, such as Kv21 channel proteins^20^, messenger RNAs in *Escherichia coli*^21^ and lipid granules in yeast cells^22^. The origin of the nonergodicity in live cells has remained elusive^20^,^21^,^22^,^23^,^24^. The immobile trajectories may have an important role in determining the difference between the time- and space-averaged 〈Δ**r**^2^(*τ*)〉, because the immobile trajectories are typically included in the space-averaged 〈Δ**r**^2^(*τ*)〉, whereas in the time-averaged 〈Δ**r**^2^(*τ*)〉, one usually only samples the mobile trajectories^20^,^21^,^22^.

### Crowding effects and anomalous diffusion

In the original model of membrane diffusion^5^, the cell membrane was considered as a continuum fluid layer, which is true only if the membrane is homogenous and the diffusing particle is much larger than the surrounding membrane molecules. For AChRs, however, their size is comparable to that of the surrounding membrane proteins and lipids, and their motion is hindered by the direct interactions with the surrounding macromolecules. In a crowded molecular solution, a tracer molecule faces a heterogeneous environment and its MSD is no longer a simple linear function of *τ*. Instead, the MSD often exhibits a sub-diffusive behaviour with 〈Δ**r**^2^(*τ*)〉∼*τ*^*α*^, where *α*<1 (refs 15, 24, 25). Such anomalous diffusion has been observed in a variety of dense fluid systems, such as colloidal diffusion near its glass transition^26^,^27^ and over an external random potential^28^.

Membrane proteins in live cells were also found to exhibit anomalous diffusion^7^,^11^,^20^,^29^. Because of the limited number and time span of the protein trajectories obtained, the measured MSD in some previous studies, however, only revealed a sub-diffusive regime without showing a crossover to the long-time diffusion. Some of the measurements also suffered relatively large statistical uncertainties at large delay times *τ*. The MSD shown in Fig. 3 clearly reveals a crossover behaviour from sub-diffusion to long-time diffusion with the crossover time *τ*_L_≃4 s. In the long-time diffusion regime as shown in the inset of Fig. 3, the measured MSD remains as a linear function of *τ* up to the longest tracking time 160 s, indicating that the membrane is very fluidic for AChRs. During this time, the AChRs diffuse more than 6 μm (or about 860 times of their own diameter) and no permanent fence is found at this length scale to confine the motion of AChRs.

In the short-time sub-diffusion regime (*τ*<4*s*), the measured 〈Δ**r**^2^(*τ*)〉 decreases with decreasing *τ* and reaches an asymptotic value 〈Δ**r**^2^〉_0_≃24.4 × 10^−3^ μm^2^ for the mobile AChRs. The value of 〈Δ**r**^2^〉_A_ for the immobile AChRs is about half the value for the mobile AChRs with 〈Δ**r**^2^〉_A_≃12.4 × 10^−3 ^μm^2^. At the *τ*→0 limit, the measured MSD becomes the mean square fluctuation, 〈Δ**r**^2^〉_0_=∑_*i*_(〈Δ**r**^2^〉_0_)_*i*_, which is a sum of (〈Δ**r**^2^〉_0_)_*i*_ from all independent fluctuation sources *i*. By subtracting out the background noise, 〈Δ**r**^2^〉_B_≃3.11 × 10^−3 ^μm^2^, from the immobile QDs, which are physically attached to the glass substrate, we find the immobile AChRs jiggle in a typical range *R*_0_=[〈Δ**r**^2^〉_A_−〈Δ**r**^2^〉_B_]/4]^1/2^≃48.2 nm. This 48.2-nm-ranged jiggling may result from the agitation of the underlying cortical actin network, which the immobile AChRs are bound to (see more discussions below). With this understanding, one may define the net MSD of the mobile AChRs without the influence of independent agitations of the actin network as (ref. 30), 〈Δ**r**^2^(*τ*)〉_AChR_=〈Δ**r**^2^(*τ*)〉−〈Δ**r**^2^〉_A_, which differs from the measured 〈Δ**r**^2^(*τ*)〉 in Fig. 3 only in the small-τ range (*τ*≲0.5* *s). The resulting 〈Δ**r**^2^(*τ*)〉_AChR_ still goes as *τ*^*α*^, but the value of *α* varies in a narrower range 0.7–0.9 with a typical value *α*≈0.8. We note that the above analysis can only remove the effect of independent agitations from the actin network and the correlated effect with the cortical network still remains in the measured MSD of the mobile AChRs.

Theoretical models of anomalous diffusion of membrane proteins have considered the effects of diffusion obstruction by permanent or transient obstacles and confinement by transient binding of diffusing proteins to a hierarchy of traps^7^,^15^,^25^,^31^. In the latter case, the time that the protein molecules are confined in the traps was assumed to have a power-law distribution^15^,^24^,^32^, which gives rise to a non-converging mean trapping time. While these models can predict certain aspects of anomalous diffusion, such as the sub-diffusion exponent *α*, the present experiment reveals some new features of membrane diffusion, which have not been considered in the previous models. The new features of membrane diffusion include the persistent exponential tail in the measured *P*(Δ*x*′) (and *P*(Δ*y*′)), which is invariant with delay time *τ*, and a crossover to apparently normal diffusion (in terms of MSD) at long-delay times (*τ*>4*s*) but with non-Gaussian statistics. One could introduce a crossover to normal diffusion by assuming that the trapping time of the protein molecules has an upper bound at equilibrium and thus their correlation time is finite. In this case, the protein trajectories would eventually be randomized at the long-time limit, and their displacement Δ*x*′ (and Δ*y*′) would follow the Gaussian statistics. Therefore, a new crossover mechanism is needed in order to explain the non-Gaussian diffusion dynamics of AChRs on live cell membrane in both the short- and long-time regimes.

### Dynamic heterogeneity and non-Gaussian statistics

Figure 5 clearly demonstrates that the lateral motion of AChRs on the live cell membrane does not follow the Gaussian statistics. For the first time, we have obtained a universal PDF with its amplitude varied by more than three decades. With such a large number of statistics, we are able to pin down the functional form of *P*(Δ*x*′) (and *P*(Δ*y*′)). The fit shown in Fig. 5 (red solid line) reveals that the measured *P*(Δ*x*′) has a simple exponential form, *P*(Δ*x*′)≃exp (−*β*|Δ*x*′|), with *β*=1.4 (which is a straight line in the semi-log plot). The obtained exponential PDFs are found to be universal independent of delay time *τ*, the measured value of *D*_L_, and the origin and cultured days of the cells.

In fact, the observed exponential form of *P*(Δ*x*′) is directly linked to the dynamic heterogeneity in the diffusion coefficient. Assuming that the entire ensemble of mobile AChRs can be divided into independent subgroups, and that each subgroup obeys the Gaussian statistics with a diffusion coefficient *δ*:


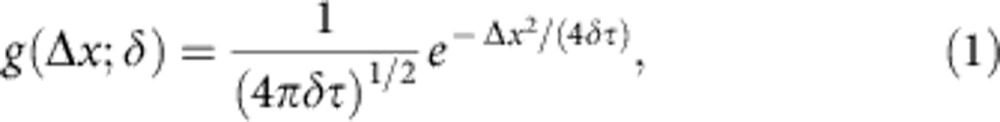


and let *δ* have an exponential-like distribution, *f*_0_(*δ*)=(1/*D*_L_)exp(−*δ*/*D*_L_), where *D*_L_ is the mean value of *δ* measured in the inset of Fig. 3. The ensemble averaged *P*(Δ*x*′) then takes the form,


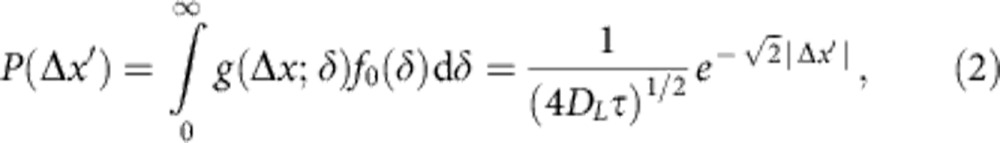


where Δ*x*′=Δ*x*/(2*D*_L_*τ*)^1/2^ is the normalized displacement. Equation (2) thus explains the exponential PDF shown in Fig. 5. The predicted decay rate 
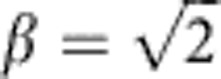
 is in excellent agreement with the measured *β*=1.4.

To further test equation (2), we directly measure the ‘instantaneous' diffusion coefficient *δ*=〈Δ**r**^2^(*τ*)〉_*t*_/(4*τ*) with the delay time set at *τ*=1 s and the averaging time *t*=4.2 s, above which the measured MSD becomes diffusive (see Fig. 3). Figure 6 shows the measured PDF *f*(*δ*′) of the normalized diffusion coefficient *δ*′=*δ*/*D*_L_ for three groups of cells under different culture conditions. The measured PDFs for the cells from different embryos or cultured on different days all collapse onto a single master curve, once the normalized *δ*′ is used in the plot. They have a universal shape with a sharp peak for small values of *δ*′ followed by an exponential-like tail (red solid line). Figures 5 and 6 thus confirm the theoretical prediction in equation (2).

Because of sampling fluctuations, the measured *δ*′ (or δ) will have its own distribution *f*(*δ*′) even for Brownian diffusion without any dynamic heterogeneity^33^,^34^. The blue dashed line in Fig. 6 shows the measured *f*(*δ*′) for the silica spheres undergoing normal Brownian diffusion, which is a narrowly peaked function with its most probable value at *δ*′≃1. It is found that the measured *f*(*δ*′) for Brownian diffusion obeys the *χ*^2^-distribution, which depends sensitively on the number 2*N* of degrees of freedom for the statistical variable *δ*′ (see Supplementary Fig. 4 for more details). In our case, we have 2*N*=8, where *N*=(*t*−*τ*_0_)/*τ*=4 and *τ*_0_=0.2 s is the sampling time used in the experiment. Compared with Brownian diffusion, the measured *f*(*δ*′) for the AChRs reveals a heavier tail with many AChR trajectories having large values of *δ*′. In addition, the measured *f*(*δ*′) for the AChRs is found to be insensitive to the change of 2*N* (see Supplementary Fig. 5 for more details). These findings further confirm that the exponential-like distribution of AChR's diffusion coefficient, as shown in Fig. 6, has its own dynamic origin and does not result from the sampling statistics (see Supplementary Discussion for more discussions).

## Discussion

Figure 6 reveals that the AChRs on live cell membrane have an exponential-like distribution in their diffusion coefficient *δ*, even though they are structurally identical. There are two possible causes for the observed dynamic heterogeneity in *δ*. One is that the AChRs form equilibrium clusters (or domains) with the surrounding proteins/lipids; and the other is that the motion of AChRs involves some active (non-equilibrium) process with a long correlation time to which the central limit theorem does not apply. There are several hypotheses in the literature on equilibrium membrane organization. One is the lipid raft model, which conceives the membrane to be compartmentalized by cholesterol organized glycolipoprotein nano-domains^3^. The typical size of the lipid rafts was estimated as 26±13 nm^35^ and they float freely in the membrane bilayer^36^. However, our results in Figs 5 and 6 cannot be explained by these features of the lipid rafts. If AChRs diffuse together with the lipid rafts, the difference in raft size is not enough to produce an exponential distribution of *δ*. This is because *δ* scales with the domain size *a* as ln(1/*a*) (ref. 5), which is essentially a constant for a moderately narrow distribution of raft size. As a result, the PDF *P*(Δ*x*′) for lipid rafts of similar size should be of Gaussian form at the limit of long delay times, as they diffuse on the membrane at equilibrium with only a finite correlation time. In fact, this argument also applies to other models of membrane organization involving phase separation and critical fluctuations in membrane at equilibrium^37^,^38^.

Another hypothesis is the picket-fence model^7^,^11^,^12^, which envisions that the membrane is compartmentalized by cortical actin ‘fences' and anchored transmembrane protein ‘pickets.' For short times, the motion of membrane proteins and lipids is transiently confined in the corrals made of the protein pickets. Over long times, the proteins and lipids can hop among different corrals following a thermal activation process. Although this model of hop diffusion can qualitatively explain some previous SPT results, it contains two key assumptions that are inconsistent with the findings of the present experiment. First, the model assumes that the corrals are quasi-periodic with a size ranging from 32 to 230 nm depending on the cell type^7^,^11^,^12^. As mentioned above, it is difficult to produce an exponential *f*(*δ*) with motion confined by corrals with a narrow size distribution. Second, the model assumes that the hopping of membrane molecules among different corrals is made by thermal fluctuations, an equilibrium process with a finite correlation time which is unlikely to produce the non-Gaussian statistics shown in Fig. 5.

On the basis of the above experimental results, we propose a dynamic picket-fence model involving slow-active remodelling of the cortical actin network to explain the observed dynamic heterogeneity. In this model, we postulate that the anchored transmembrane proteins (both immobile and transiently confined proteins) have a dominant role in determining the diffusion dynamics of other (mobile) membrane molecules. We find that 36% of the AChRs, on average, are immobile during the experimental observation time (10–15 min). For other transmembrane proteins with stronger interactions with the cortical actin network, this ratio may be even larger. Due to the abundance of membrane proteins^2^, the anchored proteins can form a continuous random network, partitioning the membrane into domains (corrals) of various sizes. Within each corral, the motion of the membrane molecules is strongly hindered by the rigid boundary of the protein network, giving rise to a local diffusion coefficient *δ*, which is strongly influenced by the size of the corral. Because the protein network on the membrane is anchored to the underlying cortical actin network, the two networks should share the same topological structure and dynamics. Without external stimulations, the protein network on the membrane will be randomly orientated having a large variety of meshes (corrals) of different sizes^39^. For short times, the mobile proteins and lipids on the membrane can move within the corrals, and over long times they also move between different corrals as the network remodels.

Our hypothesis has important biological implications, as it provides a mechanism of membrane organization for live cells to actively control the membrane fluidity and regulate the molecular transport on the membrane. It has specific predictions that can be tested in future experiments. First, the membrane protein network is not permanently stationary, as this would provide permanent barriers inhibiting the mobile proteins and lipids from moving over long distances, which is inconsistent with the measurements shown in the inset of Fig. 3. Although thermal fluctuations and ligand-binding equilibrium may provide some mobility for the protein network, these are Gaussian-like agitations and cannot produce the exponential (non-Gaussian) PDF as shown in Fig. 5. Under the dynamic picket-fence model, the dynamics of the protein network (and hence the long-time diffusion of the mobile proteins and lipids) is determined by the dynamics of the underlying cortical actin network, which is under constant active remodelling^40^,^41^,^42^,^43^. The slow-active remodelling of the cortical network (and hence the protein network) is caused by the activity of molecular motors (for example, myosin II motors) and other non-equilibrium cellular processes^44^,^45^, and thus is capable of producing fluctuations with a long correlation time, to which the central limit theorem does not apply.

Second, the long-time non-Gaussian statistics shown in Fig. 5 should be a universal behaviour for all mobile molecules on the membrane including lipids and lipid-tethered proteins on the outer leaflet of the membrane, which do not have direct interactions with the underlying cortical actin network. In a recent experiment (W. He *et al*., manuscript in preparation), we studied the lateral motion of ganglioside GM1, which is a glycosphingolipid residing on the outer leaflet of the *Xenopus* muscle-cell membrane. The GM1s was found to have a similar non-Gausian behaviour as that of the AChRs. Finally, because the non-Gaussian dynamics of the membrane molecules is regulated by the active remodelling of the cortical actin network, it will change sensitively with the dynamics of the cortical network. Various drug manipulations of the actin network, such as depletion of adenosine triphosphate and inhibition of myosin II motors, may be used to further test this prediction.

## Methods

### Cell culture

The AChR is a cation-selective, ligand-gated ion channel and consists of five subunits with diameter *d*≃7 nm. It is an integral membrane protein that responds to the binding of acetylcholine, which is a neurotransmitter. The AChR spans the membrane of muscle cells with most of its mass in the extracellular space^46^. *Xenopus* muscle cells are dissected from myotomes of the fertilized *Xenopus* embryos developed at the stages 20–22, following the protocol described in ref. 47. The dissected muscle cells are seeded on the glass cover slips coated with Entactin, Collagen-IV, and Laminin (ECL, purchased from Upstate Co.), which are immersed in a culture medium composed of 88% Steinberg's solution, 10% L-15 medium (purchased from Leibovitz Co.), 1% foetal bovine serum and 1% penicillin/streptomycin/gentamincin^47^. The muscle-cell cultures are maintained at 23 °C and can be stored for 3 weeks if they are not contaminated.

### QD labelling

To track the AChRs on a live muscle cell membrane, the individual AChRs are labelled by bright and photostable fluorescent QDs^8^,^17^. This is achieved by first labelling the AChRs with biotinylated α-bungarotoxin (biotin-BTX, purchased from Invitrogen Co.) for 10 min. The cells are then washed with the culture medium three times (5 min each). The concentration of biotin-BTX applied to the cells is adjusted according to the final labelling density of the QDs required. Typically, for a fast movie recording (for example, 80 and 5 fps), 0.5 nM biotin-BTX is used. For a slow movie recording (for example, 0.33 fps), 0.25 nM biotin-BTX is used. Lower QDs concentration is used to reduce tracking ambiguities between the consecutive images of the QDs. After repeated washing to remove unbounded biotin-BTX, 2.5 nM streptavidin-conjugated Qdot 655 solution (purchased from Invitrogen Co.) is added to the cells for 10 min after which the cells are washed with the culture medium three times (5 min each). The entire staining process requires ∼1.5 h. *Xenopus* muscle cells in the primary culture present a large area for optical observation, typically 0.05 mm^2^ on the bottom membrane and 0.002 mm^2^ on the top apical membrane. QD-labelled AChRs are abundant on the membrane of the quiescent muscle cells. This is true even for sparsely labelled samples to avoid trajectory entanglement. In the experiment, several hundreds of AChRs are tracked concurrently. For a typical bottom membrane tracking at 5 fps, 200 QDs are labelled.

Other experimental details about the optical imaging and SPT are given in Supplementary Methods.

### Data availability

The data that support the findings of this study (such as figure source data and supplementary information files) are available from the corresponding author (P.T.) upon request.

## Additional information

**How to cite this article:** He, W. *et al*. Dynamic heterogeneity and non-Gaussian statistics for acetylcholine receptors on live cell membrane. *Nat. Commun.* 7:11701 doi: 10.1038/ncomms11701 (2016).

## Supplementary Material

Supplementary Information Supplementary Figures 1-9, Supplementary Note 1, Supplementary Discussion, Supplementary Methods and Supplementary References

Supplementary Movie 1 Single particle tracking and dynamic heterogeneity of acetylcholine receptors on live muscle cell membrane. this movie shows the moving trajectory of a trans-membrane protein, acetylcholine receptor (AChR), on the membrane of a live Xenopus muscle cell after dissected for two days. The AChR is labeled by a fluorescent quantum dot. The movie was obtained using an inverted Leica microscope equipped with an Andor Ixon3 897 EMCCD camera. The movie is played in real time and the scale bar is 2 μm. To clearly reveal the details of the AChR trajectory, the image is digitally magnified by ~3 Times.

## References

[b1] Singer S. J. & Nicolson G. L. The fluid mosaic model of the structure of cell membranes. Science 175, 720–731 (1972). 4333397 10.1126/science.175.4023.720

[b2] Engelman D. M. Membranes are more mosaic than fluid. Nature 438, 578–580 (2005). 16319876 10.1038/nature04394

[b3] Lingwood D. & Simons D. Lipid rafts as a membrane-organizing principle. Science 327, 46–50 (2010). 20044567 10.1126/science.1174621

[b4] Munro S. Lipid rafts: elusive or illusive? Cell 115, 377–388 (2003). 14622593 10.1016/s0092-8674(03)00882-1

[b5] Saffman P. G. & Delbrück M. Brownian motion in biological membranes. Proc. Natl Acad. Sci. USA 72, 3111–3113 (1975). 1059096 10.1073/pnas.72.8.3111 PMC432930

[b6] Jacobson K., Sheets E. D. & Simson R. Revisiting the fluid mosaic model of membranes. Science 268, 1441–1442 (1995). 7770769 10.1126/science.7770769

[b7] Kusumi A., Umemura Y., Morone N. & Fujiwara T. in *Anomalous Transport: Foundations and Applications* (eds Klages, R., Radons, G. & Sokolov, I. M.) ch19, 545–574 (Wiley-VCH Verlag GmbH & Co. KGaA, 2008).

[b8] Bannai H., Lévi S., Schweizer C., Dahan M. & Triller A. Imaging the lateral diffusion of membrane molecules with quantum dots. Nat. Protoc. 1, 2628–2634 (2006). 17406518 10.1038/nprot.2006.429

[b9] Gelles J., Schnapp B. J. & Sheetz M. P. Tracking kinesin-driven movements with nanometre-scale precision. Nature 331, 450–453 (1988). 3123999 10.1038/331450a0

[b10] Simons K. & Sampaio J. L. Membrane organization and lipid rafts. Cold Spring Harb. Perspect. Biol. 3, a004697 (2011). 21628426 10.1101/cshperspect.a004697 PMC3179338

[b11] Ritchie K. . Detection of non-Brownian diffusion in the cell membrane in single molecule tracking. Biophys. J. 88, 2266–2277 (2005). 15613635 10.1529/biophysj.104.054106 PMC1305276

[b12] Kraft M. L. Plasma membrane organization and function: moving past lipid rafts. Mol. Biol. Cell 24, 2765–2768 (2013). 24030510 10.1091/mbc.E13-03-0165 PMC3771939

[b13] Crocker J. C. . Two-point microrheology of inhomogeneous soft materials. Phys. Rev. Lett. 85, 888–891 (2000). 10991424 10.1103/PhysRevLett.85.888

[b14] Hoffman B. D., Massiera G., Van Citters K. M. & Crocker J. C. The consensus mechanics of cultured mammalian cells. Proc. Natl Acad. Sci. USA 103, 10259–10264 (2006). 16793927 10.1073/pnas.0510348103 PMC1502445

[b15] Höfling F. & Franosch T. Anomalous transport in the crowded world of biological cells. Rep. Prog. Phys. 76, 046602 (2013). 23481518 10.1088/0034-4885/76/4/046602

[b16] Geng L., Zhang H. L. & Peng H. B. The formation of acetylcholine receptor clusters visualized with quantum dots. BMC Neurosci. 10, 80 (2009). 19604411 10.1186/1471-2202-10-80 PMC2714859

[b17] Geng L. Visualization of nicotinic acetylcholine receptor trafficking with quantum dots in Xenopus *muscle cells* PhD Thesis, HKUST (2006).

[b18] Alcor D., Gouzer G. & Triller A. Single-particle tracking methods for the study of membrane receptors dynamics. Eur. J. Neurosci. 30, 987–997 (2009). 19735284 10.1111/j.1460-9568.2009.06927.x

[b19] Walck C. Internal Report SUFPFY/9601 University of Stockholm (2007).

[b20] Weigel A. V., Simon B., Tamkun M. M. & Krapfa D. Ergodic and nonergodic processes coexist in the plasma membrane as observed by single-molecule tracking. Proc. Natl Acad. Sci. USA 108, 6438–6443 (2011). 21464280 10.1073/pnas.1016325108 PMC3081000

[b21] Golding I. & Cox E. C. Physical nature of bacterial cytoplasm. Phys. Rev. Lett. 96, 098102 (2006). 16606319 10.1103/PhysRevLett.96.098102

[b22] Jeon J. H. . *In vivo* anomalous diffusion and weak ergodicity breaking of lipid granules. Phys. Rev. Lett. 106, 048103 (2011). 21405366 10.1103/PhysRevLett.106.048103

[b23] Barkai E., Garini Y. & Metzler R. Strange kinetics of single molecules in living cells. Phys. Today 65, 29–35 (2012).

[b24] Meroz Y. & Sokolov I. M. A toolbox for determining subdiffusive mechanisms. Phys. Rep. 573, 1–29 (2015).

[b25] Saxton M. J. A biological interpretation of transient anomalous subdiffusion. I. Qualitative model. Biophys. J. 92, 1178–1191 (2007). 17142285 10.1529/biophysj.106.092619 PMC1783867

[b26] Ghosh A., Chikkadi V., Schall P. & Bonn D. Connecting structural relaxation with the low frequency modes in a hard-sphere colloidal glass. Phys. Rev. Lett. 107, 188303 (2011). 22107681 10.1103/PhysRevLett.107.188303

[b27] Hunter G. L. & Weeks E. R. The physics of the colloidal glass transition. Rep. Prog. Phys. 75, 066501 (2012). 22790649 10.1088/0034-4885/75/6/066501

[b28] Hanes R. D., Dalle-Ferrier C., Schmiedeberg M., Jenkinsa M. C. & Egelhaaf S. U. Colloids in one dimensional random energy landscapes. Soft Matter 8, 2714–2723 (2012).

[b29] Smith P. R., Morrison I. E., Wilson K. M., Fernández N. & Cherry R. J. Anomalous diffusion of major histocompatibility complex class I molecules on heLa cells determined by single particle tracking. Biophys. J. 76, 3331–3344 (1999). 10354459 10.1016/S0006-3495(99)77486-2 PMC1300303

[b30] Martin D. S., Forstner M. B. & Käs J. A. Apparent subdiffusion inherent to single particle tracking. Biophys. J. 83, 2109 (2002). 12324428 10.1016/S0006-3495(02)73971-4 PMC1302299

[b31] Soula H., Caré B., Beslon G. & Berry H. Anomalous versus slowed-down Brownian diffusion in the ligand-binding equilibrium. Biophys. J. 105, 2064–2073 (2013). 24209851 10.1016/j.bpj.2013.07.023 PMC3824720

[b32] Wong I. Y. . Anomalous diffusion probes microstructure dynamics of entangled F-actin networks. Phys. Rev. Lett. 92, 178101 (2004). 15169197 10.1103/PhysRevLett.92.178101

[b33] Bauer M., Valiullin R., Radons G. & Kärger J. How to compare diffusion processes assessed by single-particle tracking and pulsed field gradient nuclear magnetic resonance. J. Chem. Phys. 135, 144118 (2011). 22010709 10.1063/1.3647875

[b34] Heidernätsch M., Bauer M. & Radons G. Characterizing N-dimensional anisotrpic Brownian motion by the distribution of diffusivities. J. Chem. Phys. 139, 184105 (2013). 24320252 10.1063/1.4828860

[b35] Pralle A., Keller P., Florin E. L., Simons K. & Hörber J. K. Sphingolipid-cholesterol rafts diffuse as small entities in the plasma membrane of mammalian cells. J. Cell Biol. 148, 997–1008 (2000). 10704449 10.1083/jcb.148.5.997 PMC2174552

[b36] Simons K. & Ehehalt R. Cholesterol, lipid rafts, and disease. J. Clin. Invest. 110, 597–603 (2002). 12208858 10.1172/JCI16390 PMC151114

[b37] Veatch S. L. . Critical fluctuations in plasma membrane vesicles. ACS Chem. Biol. 3, 287–293 (2008). 18484709 10.1021/cb800012x

[b38] Heberle F. A. . Bilayer thickness mismatch controls domain size in model membranes. J. Am. Chem. Soc. 135, 6853–6859 (2013). 23391155 10.1021/ja3113615

[b39] Novikov D. S., Fieremans E., Jensen J. H. & Helpern J. A. Random walks with barriers. Nat. Phys. 7, 508–514 (2011). 21686083 10.1038/nphys1936 PMC3114643

[b40] Gowrishankar K. . Active remodeling of cortical actin regulates spatiotemporal organization of cell surface molecules. Cell 149, 1353–1367 (2012). 22682254 10.1016/j.cell.2012.05.008

[b41] Luo W. W. . Analysis of the local organization and dynamics of cellular actin networks. J. Cell. Biol. 202, 10571073 (2013). 10.1083/jcb.201210123 PMC3787384 24081490

[b42] Guo M. . Probing the stochastic, motor-driven properties of the cytoplasm using force spectrum microscopy. Cell 158, 822–832 (2014). 25126787 10.1016/j.cell.2014.06.051 PMC4183065

[b43] Parry B. R. . The Bacterial cytoplasm has glass-like properties and is fluidized by metabolic activity. Cell 156, 1–12 (2014). 10.1016/j.cell.2013.11.028 PMC3956598 24361104

[b44] Brangwynne C. P., Koenderink G. H., MacKintosh F. C. & Weitz D. A. Cytoplasmic diffusion: molecular motors mix it up. J. Cell. Biol. 183, 583–587 (2008). 19001127 10.1083/jcb.200806149 PMC2582900

[b45] Prost J., Jülicher F. & Joanny J. F. Active gel physics. Nat. Phys 11, 111–117 (2015).

[b46] Unwin N. Neurotransmitter action: opening of ligand-gated ion channels. Cell Suppl. 72, Suppl 31–41 (1993). 10.1016/s0092-8674(05)80026-1 7679054

[b47] Peng H. B., Baker L. P. & Chen Q. Tissue culture of *Xenopus* neurons and muscle cells as a model for studying synaptic induction. Methods Cell Biol. 36, 511–526 (1991). 1811149 10.1016/s0091-679x(08)60294-0

