# Supplementary material for: Dynamic heterogeneity and non-Gaussian statistics for acetylcholine receptors on live cell membrane
Source: Nat Commun. 2016 May 26;7:11701. doi: 10.1038/ncomms11701 (PMC4894960; doi:10.1038/ncomms11701)
Supplement: Supplementary Information — Supplementary Figures 1-9, Supplementary Note 1, Supplementary Discussion, Supplementary Methods and Supplementary References [file ncomms11701-s1.pdf]

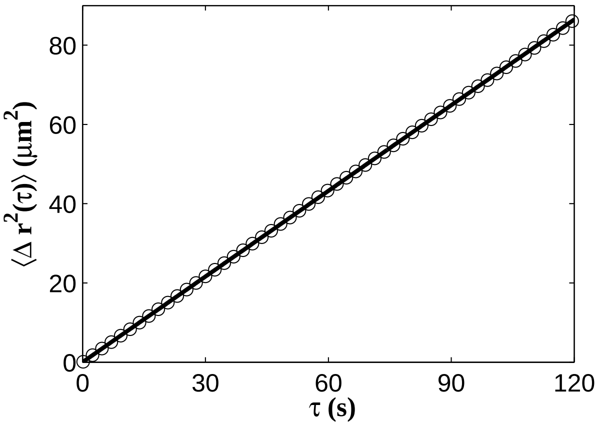

Supplementary Figure 1. Measured MSD $\langle \Delta r^2(\tau) \rangle$ as a function of delay time τ for the silica spheres diffusing over a flat surface. The solid line is a linear fit to the data points.

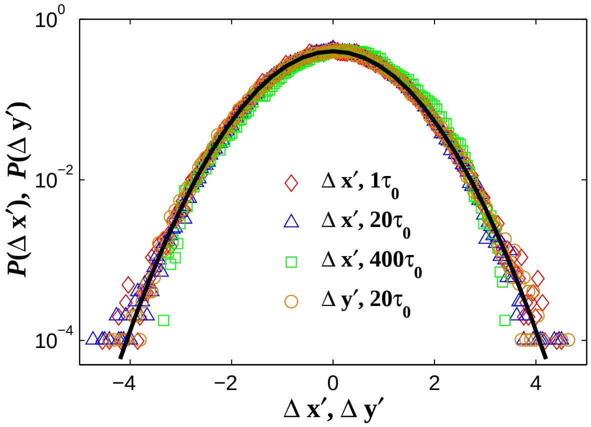

Supplementary Figure 2. (Color online) Measured probability density functions (normalized histograms) $P(\Delta x')$ and $P(\Delta y')$ of the normalized displacements $\Delta x'(\tau)$ and $\Delta y'(\tau)$ for the silica spheres. Data are obtained under different conditions: (i) $\Delta x'(\tau)$ with $\tau = 1\tau_0$ (red diamonds), $20\tau_0$ (blue triangles), $400\tau_0$ (green squares), and (ii) $\Delta y'(\tau)$ with $\tau = 20\tau_0$ (orange circles). Here $\tau_0 = 0.153$ s is the smallest sampling time used in the experiment. The black solid line shows the standard normal distribution given in Supplementary Eq. (2).

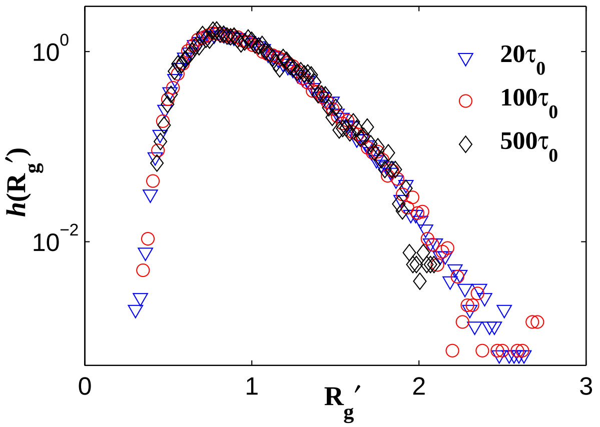

Supplementary Figure 3. (Color online) Measured probability density function (normalized histogram) $h(R'_g)$ of the normalized radius of gyration $R'_g(\tau)$ for different values of τ: $20\tau_0$ (blue triangles), $100\tau_0$ (red circles), and $500\tau_0$ (black diamonds). Here $\tau_0 = 0.153$ s is the smallest sampling time used in the experiment.

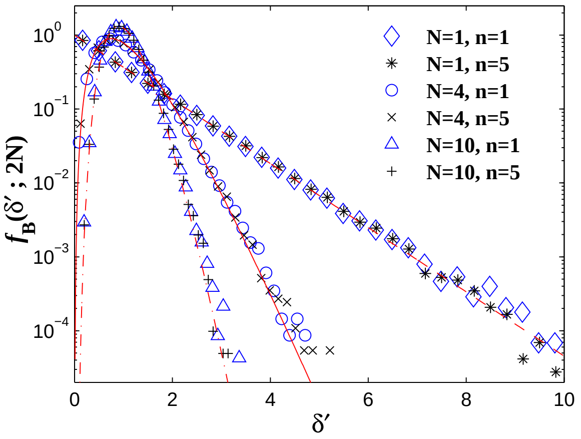

Supplementary Figure 4. (Color online) Measured probability density function (normalized histogram) $f_B(\delta'; 2N)$ of the normalized diffusion coefficient δ' for the silica spheres. Data are obtained for different degrees of freedom $2N$ and delay times $\tau = n\tau_0$: $N = 1$, $n = 1$ (blue diamonds), $N = 1$, $n = 5$ (black asterisks), $N = 4$, $n = 1$ (blue circles), $N = 4$, $n = 5$ (black crosses), $N = 10$, $n = 1$ (blue triangles), and $N = 10$, $n = 5$ (black pluses). Here $\tau_0 = 0.153$ s is the smallest sampling time used in the experiment. The red solid and dash-dotted lines show, respectively, the χ^2-distribution in Supplementary Eq. (10) with $N = 4$ and $N = 10$. The red dashed line shows the χ^2-distribution given in Supplementary Eq. (11) with $N = 1$.

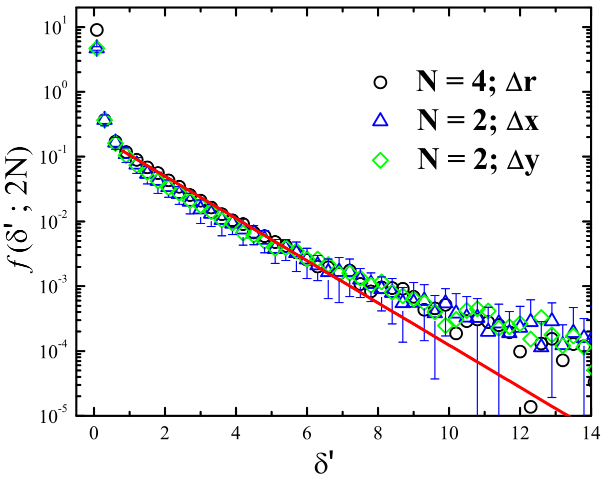

Supplementary Figure 5. Measured PDF $f(\delta')$ of the normalized diffusion coefficient $\delta' = \delta/D_L$ for the mobile AChR trajectories with different values of the experimental sampling number: $2N = 8$ (black circles), $2N = 4$ (blue triangles), and $2N = 4$ (green diamonds). Data are obtained from 10 cells cultured for three days. The red solid line shows the same exponential function, $f(\delta') \simeq 0.22 \exp(-0.75\delta')$, as that in Fig. 6 of the main text.

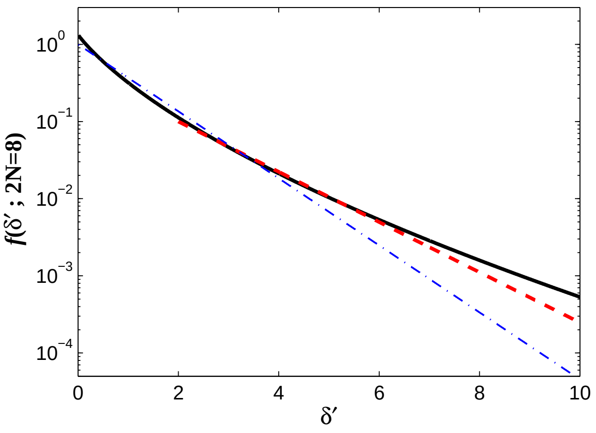

Supplementary Figure 6. (Color online) Calculated $f(\delta'; 2N = 8)$ using Supplementary Eq. (14) (black solid line). The blue dash-dotted line shows the intrinsic distribution function $f_0(\delta')$ in Supplementary Eq. (12). The red dashed line shows the exponential function, $f(\delta') = 0.44 \exp(-0.75\delta')$.

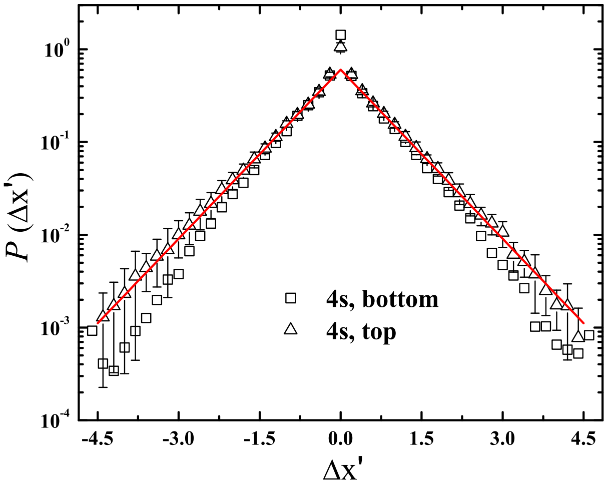

Supplementary Figure 7. Measured PDF $P(\Delta x')$ of the normalized displacement $\Delta x'$ for the trajectories of mobile AChRs on the lower portion (facing the substrate) of the membrane (squares) and on the upper portion (away from the substrate) of the membrane (triangles). Data are obtained from 10 cells with $\tau = 4$ s. The error bars show the standard deviation of the triangles averaged over 10 cells. The red solid line shows the same exponential function, $P(\Delta x') \simeq 0.6 \exp(-1.4|\Delta x'|)$, as that shown in Fig. 5 of the main text.

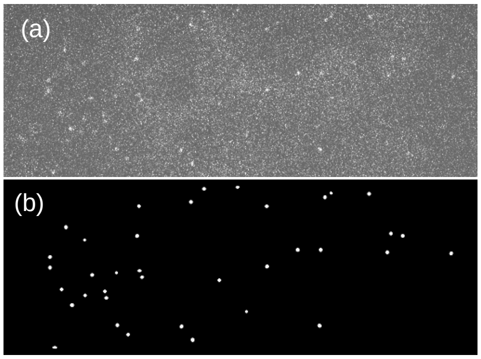

Supplementary Figure 8. Comparison between the raw and processed images of QD-labeled AChRs. (a) A raw image $I_0(x, y)$ of QDs obtained at the sampling rate 80 fps with an exposure time of 10 ms. (b) Final image $I_2(x, y)$ of QDs obtained after the image processing as described in the text.

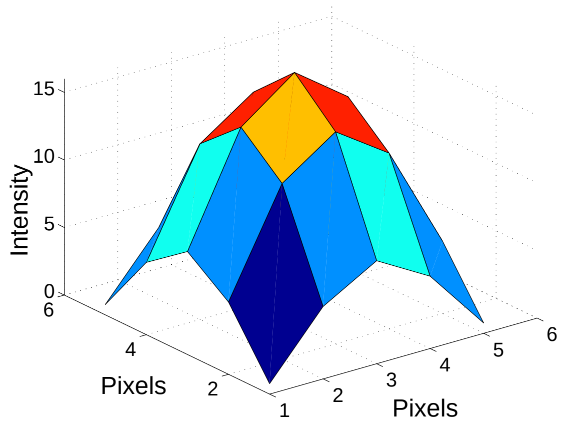

Supplementary Figure 9. (Color online) An example of the shape matrix S for a single QD, which is obtained by averaging 25 QD imagines sampled at 80 fps.

Supplementary Note: Additional experimental results

Basic statistical properties of Brownian trajectories. To further understand the dynamics of AChRs on the live cell membrane, we carried out a parallel study of diffusion dynamics of model colloidal particles and compare the main features of Brownian dynamic with those of the AChRs. The colloidal particles used in the experiment are uniform silica spheres of 1.57 μm in diameter, which have SiO^- groups on the surface. The silica spheres are dispersed in water and they settle down by gravity near the bottom water-glass interface of the sample cell. The sample cell is viewed under an inverted microscope, and the two-dimensional (2D) motion of the silica spheres is recorded with a digital camera. The same single particle tracking program used for AChRs is used to determine the particle position $r(t)$ at time t, and the particle trajectories are constructed from the consecutive images.

This is a well-characterized colloidal system, and the interactions and dynamics in the concentrated suspension of silica spheres has been studied recently [1]. Here we focus on the single particle diffusion in the dilute suspension. From the particle trajectories, we obtain the single-particle mean square displacement (MSD), $\langle \Delta r^2(\tau) \rangle$, as a function of delay time τ, where $\Delta \mathbf{r}(\tau) = \mathbf{r}(\tau + t) - \mathbf{r}(t)$. For 2D diffusion, one has [2]

$$\langle \Delta r^2(\tau) \rangle = 4D_0\tau, \tag{1}$$

where $D_0 = k_BT/(3\pi\eta d)$ is the Stokes-Einstein free diffusion coefficient with η being the fluid viscosity and $d = 1.57$ μm the diameter of the silica spheres. Supplementary Fig. **1** shows the measured $\langle \Delta r^2(\tau) \rangle$ as a function of delay time τ for the silica spheres. The data is well described by Supplementary Eq. (1) (solid line), from which we obtain $D_0 = 0.18$ $\mu m^2/s$ for the silica spheres.

Another important property of Brownian diffusion is that the probability density function (PDF) $P(\Delta x')$ of the normalized displacement $\Delta x' = \Delta x/(2D_0\tau)^{1/2}$, where $\Delta x(\tau)$ is the $x-$component of $\Delta \mathbf{r}(\tau)$, obeys the standard normal distribution:

$$P(\Delta x')d\Delta x' = \frac{1}{\sqrt{2\pi}} e^{-\Delta x'^2/2} d\Delta x'. \tag{2}$$

The PDF $P(\Delta y')$ has the same Gaussian form as the diffusion is isotropic. Supplementary Fig. **2** shows the measured PDFs (normalized histograms) $P(\Delta x')$ and $P(\Delta y')$ of the normalized displacements $\Delta x'(\tau)$ and $\Delta y'(\tau)$ for different values of τ. All the data points collapse on a single master curve, which is well described by the standard normal distribution given in Supplementary Eq. (2).

Further statistical analysis of Brownian trajectories. There are two more useful measures to characterize the trajectories of Brownian diffusion. One is the radius of gyration R_g, which is defined as

$$R_g^2(\tau) = \frac{1}{N}\sum_{i=1}^{N} [(x_i - \langle x \rangle)^2 + (y_i - \langle y \rangle)^2], \tag{3}$$

where x_i and y_i are the $x-$ and $y-$components of the position of each trajectory step and $\langle x \rangle$ and $\langle y \rangle$ are their mean values. Physically, R_g is a measure of the size of the trajectory containing N time steps with the total duration $\tau = N\tau_0$. Here τ_0 (= 0.153 s) is the smallest sampling time used in the experiment. Supplementary Eq. (3) can be re-written in terms of displacement squares:

$$R_g^2(\tau) = \frac{1}{N}\sum_{j=1}^{N-1} \frac{a_j^2 + b_j^2}{(N-j)(N-j+1)}, \tag{4}$$

where

$$a_j = \sum_{i=j}^{N-1} (N-i)\Delta x_i, \tag{5}$$

and

$$b_j = \sum_{i=j}^{N-1} (N-i)\Delta y_i. \tag{6}$$

Here $\Delta x_i = x_{i+1} - x_i$ and $\Delta y_i = y_{i+1} - y_i$ are the $x-$ and $y-$components of the displacement in each successive time step. For Brownian diffusion, Δx_i and Δy_i for different time steps are all independent. By employing $\langle \Delta x_i \Delta x_j \rangle_{i \neq j} =$

$\langle \Delta x_i \rangle \langle \Delta x_j \rangle = 0$ and $\langle \Delta x_i^2 \rangle = 2D_0\tau_0$ (the same is true for the $y-$component), we find

$$\langle R_g^2(\tau) \rangle = \frac{2D_0(N^2-1)\tau_0}{3N} \simeq \frac{2D_0\tau}{3}. \tag{7}$$

To compare trajectories with different values of τ (or N), we use the normalized radius of gyration $R_g' = R_g/\langle R_g^2 \rangle^{1/2}$. Supplementary Fig. **3** shows the measured PDF (normalized histogram) $h(R_g')$ of the normalized radius of gyration $R_g'(\tau)$ for different values of τ. All the data points collapse onto a single master curve, once the normalized R_g' is used. For silica spheres undergoing Brownian diffusion, their $h(R_g')$ is asymmetric and has a peak at the most probable value $R_g' \simeq 0.78$, which is slightly smaller than the mean value $R_g' = 1$. Because the summation in Supplementary Eq. (4) contain cross terms of $\Delta x_i \Delta x_j$ and $\Delta y_i \Delta y_j$, it is difficult to obtain an analytic form of $h(R_g')$.

The other measure to characterize the Brownian trajectories is the "instantaneous" diffusion coefficient

$$\delta = \frac{\langle \Delta \mathbf{r}^2(\tau) \rangle_t}{4\tau}, \tag{8}$$

where t is the averaging time. All the displacements $\Delta \mathbf{r}$ in the average have the same delay time $\tau < t$ and they do not overlap with each other in time. The number of the terms in the average is $2N = 2(t-\tau_0)/\tau$. For Brownian diffusion, the $2N$ terms of $\Delta x(\tau)$ and $\Delta y(\tau)$ in the average are all independent and thus they follow the same Gaussian distribution as shown in Supplementary Eq. (2). It is straightforward to show that for Brownian diffusion one has

$$\langle \delta \rangle = \frac{\langle [\langle \Delta x^2(\tau) \rangle_t + \langle \Delta y^2(\tau) \rangle_t] \rangle}{4\tau} = D_0. \tag{9}$$

To quantitatively characterize the dynamic heterogeneity of particle trajectories, we introduce the probability density function (normalized histogram) $f(\delta'; 2N)$ of the normalized diffusion coefficient $\delta' = \delta/\langle \delta \rangle$. In the main text, we used the notation $f(\delta')$ ($\equiv f(\delta'; 2N)$) for simplicity. For Brownian diffusion, because $2N\delta'$ contains a sum of squares of $2N$ independent variables $\Delta x(\tau)/(4\tau\langle\delta\rangle)$ and $\Delta y(\tau)/(4\tau\langle\delta\rangle)$ both with the standard normal distribution, $f(\delta'; 2N)$ obeys the χ^2-distribution with $2N$ degrees of freedom [3, 4]. By changing the variable from $2N\delta'$ to δ', we find $f(\delta'; 2N)$ has the form

$$f_B(\delta'; 2N) = 2N \frac{(2N\delta')^{(N-1)} e^{-N\delta'}}{2^N \Gamma(N)}, \tag{10}$$

where $\Gamma(N)$ is the Γ-function, and prefactor $2N$ is a normalization factor. The subscript B is used to denote Brownian diffusion.

Supplementary Fig. **4** shows the measured $f_B(\delta'; 2N)$ for the silica spheres undergoing normal Brownian diffusion with different degrees of freedom $2N$ and delay times $\tau = n\tau_0$. It is seen that the measured $f_B(\delta'; 2N)$ is well-described by the χ^2-distribution (red solid, dash-dotted, and dashed lines), which is uniquely determined by the value of $2N$ and is independent of n. The width of the distribution decreases with increasing values of $2N$. Physically, the χ^2-distribution tells us what the minimum variations one can get for the normalized diffusion coefficient δ' (or $2N\delta'$), which is obtained by averaging over a finite number of $2N$ samples. This conclusion only applies to the normal Brownian diffusion (without any dynamic heterogeneity), where all the squared displacement terms are independent and obey the Gaussian statistics. As a result, the variations of the measured δ' shown in Supplementary Fig. **4** result solely from the statistical fluctuations due to the finite number of $2N$ samples used in obtaining the value of δ'. When $2N$ is very large, the χ^2-distribution will become very narrow and thus the measured δ' will have little fluctuations.

Supplementary Fig. **4** thus establishes a lower bound for the statistical variations of the independent random variable δ' with $2N$ degrees of freedom. It is seen that the functional form of the measured $f_B(\delta'; 2N)$ for normal diffusion changes sensitively with the value of $2N$. In particular, when $2N = 2$, the χ^2-distribution in Supplementary Eq. (10) becomes a simple exponential function [4, 5]

$$f_B(\delta'; 2N = 2) = e^{-\delta'}, \tag{11}$$

which is shown by the red dashed line in Supplementary Fig. **4**. Note that the exponential distribution in Supplementary Eq. (11) only applies to a special case, which can be achieved when the "instantaneous" diffusion coefficient δ' (or δ) is obtained without any temporal average (so that temporal sampling fluctuations are very large) and the only sample average received in δ' is from the two-dimensional sampling ($2N = 2$). In this case, the measured $f_B(\delta'; 2N = 2)$ has a heavier tail, as shown in Supplementary Fig. **4**.

Supplementary Discussion

In the experiment for AChRs, however, we use a finite time window for sample averaging. In this case, we choose the delay time τ in Supplementary Eq. (8) to be the shortest one in the diffusion regime, so that the diffusion coefficient is meaningful. For the fixed value of τ, the pure sampling fluctuations in the measured δ' are minimized if a longer averaging time t (or a large value of $2N$) is used. However, if the averaging time t is too long, the intrinsic fluctuations resulting from the dynamic heterogeneity that we want to study here will be averaged out in the measured δ'. In Fig. 6 of the main text, a compromised value of $2N = 8$ is chosen (with $N = (t - \tau_0)/\tau = 4$, where $\tau_0 = 0.2$ s is the sampling time used in the experiment), so that the sampling fluctuations in the measured δ' are minimized and at the same time the measured $f(\delta'; 2N)$ can clearly reveal the non-Gaussian fluctuations of the measured δ', resulting from the dynamic heterogeneity of the AChRs on the live cell membrane.

Supplementary Fig. **5** shows a comparison of the measured PDFs $f(\delta'; 2N)$ for the mobile AChR trajectories with three different values of $2N$. Here the "instantaneous" diffusion coefficient δ is obtained in three different ways. The black circles are obtained for $\delta = \langle \Delta r^2(\tau) \rangle_t/(4\tau)$, which are the same as those shown in Fig. 6 of the main text with $2N = 8$. The blue triangles are obtained for the displacements in the x-direction with $\delta = \langle \Delta x^2(\tau) \rangle_t/(2\tau)$. Similarly, the green diamonds are obtained for the displacements in the y-direction with $\delta = \langle \Delta y^2(\tau) \rangle_t/(2\tau)$. In the latter two cases, because the sampling of the AChR trajectories is reduced to one-dimension, the value of $2N$ is changed to $2N = 4$. It is seen that the three sets of data superimpose well with each other and are insensitive to the change of values of $2N$. Supplementary Fig. **5** thus further confirms that the exponential-like distribution in AChR's diffusion coefficient, as shown in Fig. 6 of the main text, has its own dynamic origin and does not result from the sampling statistics, as indicated in Supplementary Eq. (11). Furthermore, the superposition of the blue triangles and green diamonds suggests that the dynamic heterogeneity of the AChRs on the cell membrane is isotropic and does not have a preferred direction.

For the AChR trajectories, the measured $f(\delta'; 2N)$ is convoluted by the finite sampling distribution $f_B(\delta'; 2N)$ and intrinsic distribution $f_0(\delta')$ due to dynamic heterogeneity. Assuming $f_0(\delta')$ has the form

$$f_0(\delta') = e^{-\delta'}, \tag{12}$$

the measured $f(\delta'; 2N)$ can be written as

$$f(\delta'; 2N) = \int_0^\infty f_0(\sigma') g(\delta'/\sigma'; 2N) d\sigma', \tag{13}$$

where $\sigma' = \sigma/D_L$ and $g(\delta'/\sigma'; 2N)$ is the probability density function of the normalized diffusion coefficient δ'. Here δ' is the variable with sampling fluctuations for a given (mean) value of σ'. Using the equation $g(\delta'/\sigma'; 2N)d\delta' = f_B(\delta'/\sigma'; 2N)d(\delta'/\sigma')$, we find $g(\delta'/\sigma'; 2N) = (1/\sigma')f_B(\delta'/\sigma'; 2N)$. Supplementary Eq. (13) then becomes

$$\begin{aligned} f(\delta'; 2N) &= \int_0^\infty \frac{1}{\sigma'} e^{-\sigma'} f_B(\delta'/\sigma'; 2N) d\sigma' \\ &= \frac{2N^{\frac{N+1}{2}}}{\Gamma(N)} \delta'^{\frac{N-1}{2}} K_{N-1}(2\sqrt{N\delta'}), \end{aligned} \tag{14}$$

where $K_{N-1}(x)$ is the modified Bessel function of the second kind.

To further evaluate the convolution effect of $f_B(\delta'; 2N)$ on the measured $f(\delta'; 2N)$, we plot, in Supplementary Fig. **6**, the calculated $f(\delta'; 2N = 8)$ using Supplementary Eq. (14) (black solid line) and the intrinsic distribution function $f_0(\delta')$ in Supplementary Eq. (12) (blue dash-dotted line). Here the experimental sampling number $2N = 8$ is used in the plot. It is found that the calculated $f(\delta'; 2N = 8)$ has a shape very much like that of the measured $f(\delta')$, as shown in Fig. 6 of the main text (and in Supplementary Fig. **5**), in which the tail part of the measured $f(\delta')$ levels off from the exponential decay at large values of δ'. This is because the convolution function $(1/\sigma')f_B(\delta'/\sigma'; 2N)$ in Supplementary Eq. (14) is a sharply peaked function of σ' (δ-function-like) for small values of δ' and becomes a slow decaying function of σ' [slower than $\exp(-\sigma')$] for large values of δ'. In the common range of δ', both the calculated $f(\delta'; 2N = 8)$ and measured $f(\delta')$ can be described approximately by the same exponential function, $f(\delta') \sim \exp(-0.75\delta')$ (red dashed line), further confirming our hypothesis that the AChRs have an exponential-like distribution function in diffusion coefficient δ', as shown in Supplementary Eq. (12).

Supplementary Fig. **7** shows a comparison of the measured PDFs $P(\Delta x')$ for mobile AChRs obtained under two different sample conditions. The squares are obtained from the trajectories of mobile AChRs on the lower portion (facing the substrate) of the membrane. The triangles are obtained from the trajectories of mobile AChRs on the upper portion (away from the substrate) of the membrane. It is seen that the two sets of data superimpose well

with each other, which suggests that the non-Gaussian behavior of the measured $P(\Delta x')$ is a generic property of the AChRs and is not influenced by the narrow gap between the basal plane of the cell membrane and substrate.

Supplementary Methods

Optical imaging. The live sample cells are placed on the stage of an inverted microscope (Leica DM-IRB), and the motion of QDs (and hence AChRs) is viewed from below under the mode of fluorescent microscopy. Image sequences are recorded using an electron-multiplying charged-coupled device (EMCCD) camera (Andor Ixon3 897 back-illuminated EMCCD) and streamed to the hard drive of a host computer. A small fraction of the QD movies are obtained using an Olympus IX70 microscope equipped with a chilled-CCD camera (Hamamatsu, ORCA). The QDs (Qdot 655) are excited by an UV light with a wavelength range between 400 nm and 450 nm, and the emitted light by the QDs is selected in the wavelength range between 645 nm and 655 nm. Typical frame rates used in the movie recording are 5 fps and 80 fps, and the corresponding exposure time is 30 ms and 10 ms, respectively. The recorded images have 14 bits of gray scales and a spatial resolution of 512×512 pixels at the frame rate 5 fps and ∼500×200 pixels at 80 fps. With a 100× oil objective, the QDs in the image taken at 80 fps typically occupy 5 pixels across their diameter. From the movie of stationary QDs (stuck on a glass slide), we find their displacement over a time period of 200 s is less than 24 nm (=0.18 pixel). This result sets the accuracy of the measurement of the displacement, $\Delta\mathbf{r}(\tau) = \mathbf{r}(t+\tau) - \mathbf{r}(t)$, where $\tau \leq 180$ s is used in the experiment. During this delay time, AChRs typically move several micrometers. Each movie file contains 1000 frames at a specific frame rate, which typically yields 1000-3000 QD trajectories. For some movie files, the recording time is extended to 30 min. In this case, the exposure time is carefully controlled in order to reduce the damage to the live cell samples.

Single particle tracking. A homemade Matlab program based on the standard tracking algorithm [6] is used to determine the AChR position $\mathbf{r}(t)$ at time t, and the AChR trajectories are constructed from the consecutive images. Several specific improvements are made in order to increase the tracking accuracy of QDs. First, the raw images of QDs on a live cell membrane are quite noisy because of the random blinking of QDs and low excitation intensity and short exposure time used in the experiment to protect the live cell sample. Supplementary Fig. **8**(a) shows an example of the raw image of QDs obtained at 80 fps with an exposure time of 10 ms. In the image, individual QDs appear as florescent light spots, which are too small to be resolved by an optical microscope (the size of Qdot 655 is ∼22 nm). These florescent spots are actually the point spread function (PSF) of the QDs under the microscope system. In our optical setup (with (Leica DM-IRB), each QD typically occupies 5×5 pixels, and the size of a (square) pixel is $p_w =$ 133 nm.

There are two types of background noise in the raw image $I_0(x, y)$, as shown Supplementary Fig. **8**(a). The first type results primarily from the auto-fluoresce of the internal components of the live cell and from the camera's dark current and shot noise. The auto-fluoresce of a live cell gives rise to intensity inhomogeneities at a length scale much larger than the size of QDs, and thus can be readily identified by applying a high-pass filter to the original image. The resulting auto-fluoresce image is then subtracted out from $I_0(x, y)$. The dark current and shot noise of the camera produce some noisy light spots of size much smaller than that of QDs and a low level fluctuating background of light intensity to all pixels. This type of background noise is removed by applying a bandpass filter to the image, followed by a subtraction of a constant intensity threshold, which is set based on the intensity distribution of all pixels in the image. After these noise-reduction procedures, we obtain the image $I_1(x, y)$, which is a 512×512 matrix for a full frame image.

The second type of noise has a range of sizes similar to that of QDs and it is often intertwined with the intrinsic fluoresce fluctuations of QDs due to different reasons, such as QD clustering and/or blinking. In this case, we use the technique of template matching [7] to remove the effect of this type of noise. The template used in the experiment is the typical shape of a single QD, which is obtained by averaging 25 QD imagines from $I_1(x, y)$. Supplementary Fig. **9** shows an example of the shape of a single QD, which is well described by a 5×5 shape matrix S for the QD. With the technique of template matching, we compute the normalized cross-correlation matrix C between the image matrix $I_1(x, y)$ and the shape matrix S

$$C = \frac{(I_1 - \bar{I}_1) * (S - \bar{S})}{I_{1\sigma} S_\sigma}, \tag{15}$$

where the symbol $*$ denotes the cross-correlation operator, $\bar{I}_1$ and $\bar{S}$ are, respectively, the mean value of the matrixes I_1 and S, and $I_{1\sigma}$ and S_σ are their standard deviations. A new image $I_2(x, y)$ is generated by multiplying the resulting correlation matrix C with a proportional constant, typically ∼1000, so that the intensity of the newly obtained optical spots has a similar value as that of the original QDs.

The correlation matrix C serves for two purposes. First, for those optical spots of the candidate QDs whose intensity profile has a similar shape as the shape matrix S, their cross-correlation coefficient with S has a maximal

value, so that the newly obtained optical spots of the candidate QDs in $I_2(x, y)$ appear more uniform both in intensity and in size. Second, for those optical spots whose intensity profile is very different from the shape matrix S, their cross-correlation coefficient with S will have a small value, so that one can set up certain selection rules to remove some unwanted optical spots in $I_2(x, y)$. In this case, we carefully choose a set of intensity criteria to exclude those spots whose intensity is either too dim or too bright. Some aggregated spots with a bright area much larger than 5×5 pixels are not selected either. Supplementary Fig. **8**(b) shows an example of the final image $I_2(x, y)$ of the candidate QDs obtained after the image processing as described above. In comparison with the raw image $I_0(x, y)$, the processed image $I_2(x, y)$ becomes much cleaner and clearer than $I_0(x, y)$, and the image of the individual QDs appears more uniform both in intensity and in size. The image processing procedures discussed above improve the signal-to-noise ratio of the QD images significantly and thus help to increase the tracking accuracy of QDs. These procedures are particularly important for those images taken under the condition of short exposure times and/or low light intensities, such as the 80 fps video acquisition with the exposure time of 10 ms and a 50% reduction of UV light source.

The center of the location of each individual florescent spot is then determined from the local maximum of the cross-correlation matrix F between the processed image $I_2(x, y)$ and the standard kernel matrix G of the point spread function (PSF)

$$F = \frac{(I_2 - \bar{I}_2) * (G - \bar{G})}{I_{2\sigma} G_\sigma}, \tag{16}$$

where $\bar{I}_2$ and $\bar{G}$ are, respectively, the mean value of the matrixes I_2 and G, and $I_{2\sigma}$ and G_σ are their standard deviations. In the above, the PSF template G is approximated by an isotropic 5×5 Gaussian matrix with a standard deviation $\sigma_{PSF} = R_A/3$, where $R_A \simeq 1.22\lambda/(2N_A)$ is the Airy radius for an incident light of wavelength λ and numerical aperture N_A. In our experiment (with Leica DM-IRB), we $N_A = 1.40$, $\lambda \simeq 655$ nm, and thus $\sigma_{PSF} \simeq 95$ nm $\simeq 0.71$ pixels. The central position of the intensity profile is thus chosen as the center of the QDs (and hence AChRs). With this method we are able to obtain a repeatable tracking accuracy of ∼20 nm for the AChR trajectories.

A difficulty in the AChR tracking is the blinking effect of the QDs [8]. The florescent intensity of the QDs fluctuates constantly and becomes invisible intermittently. This blinking effect causes breakup of some continuous trajectories and thus reduce the accuracy of the long-time tracking of the AChRs. The broken trajectories can be relinked using a searching algorithm in Ref. [6]. The algorithm was originally designed to relink the broken trajectories of those colloid particles moving in and out of the observation area. It applies well here to reconnect the fragmental trajectories of QDs. To identify the next position of a QD from a previous frame, a search radius r is carefully chosen using the diffusion radius $r = (D_L \tau_0)^{1/2}$ as an estimate of the upper limit of a single step displacement, where D_L is the diffusion coefficient of the AChRs and τ_0 is the time interval between the two adjacent frames. The program then attempts to find the nearest QD within the defined radius r. If no QD is present in the searching area, the QD is missing either permanently or temporarily because of the QD blinking. A record of missing QD counts is set up in the tracking program to retrieve those missing QDs within a time period of 1 s (within 5 frames at 5 fps). This relinking procedure increases the tracking length of the AChR trajectories and thus enhances the accuracy of their long-time statistics. To further build up the statistics, we typically track AChRs from more than 30 cells cultured under the same condition, so that (1-5)$\times 10^5$ trajectories are obtained for the statistical analysis reported in the main text.

Supplementary References

[1] Ma, X. G. et al. Test of the Universal Scaling Law of Diffusion in Colloidal Monolayers. *Phys. Rev. Lett.* **110**, 078302 (2013).

[2] Reif, F. *Fundamentals of Statistical and Thermal Physics.* (McGraw-Hill, Auckland, 1985).

[3] Freedman, D., Pisani, R. & Purves, R. *Statistics (4th edition).* (W. W. Norton and Co. Inc., 2007).

[4] Bauer, M., Valiullin, R., Radons, G. & Kärger, J. How to compare diffusion processes assessed by single-particle tracking and pulsed field gradient nuclear magnetic resonance. *J. Chem. Phys.* **135**, 144118 (2011).

[5] Heidernätsch, M., Bauer, M. & Radonsa, G. Characterizing N-dimensional anisotrpic Brownian motion by the distribution of diffusivities. *J. Chem. Phys.* **139**, 184105 (2013).

[6] Crocker, J. C. & Grier, D. G. Methods of digital video microscopy for colloidal studies. *J. Colloid and Interface Science* **179**, 298-310 (1996).

[7] Bonneau, S., Cohen, L. & Dahan, M. A multiple target approach for single quantum dot tracking. *Proceedings IEEE of International Symposium on Biological Imaging* **1**, 664-667 (2004).

[8] Michalet, X. et al. Quantum dots for live cells, in vivo imaging, and diagnostics. *Science* **307**, 538-544 (2005).
